# Antigenic Variation in *Plasmodium falciparum* Malaria Involves a Highly Structured Switching Pattern

**DOI:** 10.1371/journal.ppat.1001306

**Published:** 2011-03-03

**Authors:** Mario Recker, Caroline O. Buckee, Andrew Serazin, Sue Kyes, Robert Pinches, Zóe Christodoulou, Amy L. Springer, Sunetra Gupta, Chris I. Newbold

**Affiliations:** 1 Department of Zoology, University of Oxford, Oxford, United Kingdom; 2 Department of Epidemiology, Harvard School of Public Health, Boston, Massachusetts, United States of America; 3 Weatherall Institute of Molecular Medicine, University of Oxford, John Radcliffe Hospital, Oxford, United Kingdom; 4 Department of Biology, Amherst College, Amherst, Massachusetts, United States of America; Albert Einstein College of Medicine, United States of America

## Abstract

Many pathogenic bacteria, fungi, and protozoa achieve chronic infection through an immune evasion strategy known as antigenic variation. In the human malaria parasite *Plasmodium falciparum*, this involves transcriptional switching among members of the *var* gene family, causing parasites with different antigenic and phenotypic characteristics to appear at different times within a population. Here we use a genome-wide approach to explore this process *in vitro* within a set of cloned parasite populations. Our analyses reveal a non-random, highly structured switch pathway where an initially dominant transcript switches via a set of switch-intermediates either to a new dominant transcript, or back to the original. We show that this specific pathway can arise through an evolutionary conflict in which the pathogen has to optimise between safeguarding its limited antigenic repertoire and remaining capable of establishing infections in non-naïve individuals. Our results thus demonstrate a crucial role for structured switching during the early phases of infections and provide a unifying theory of antigenic variation in *P. falciparum* malaria as a balanced process of parasite-intrinsic switching and immune-mediated selection.

## Author Summary

The malaria parasite *Plasmodium falciparum* avoids recognition and clearance by the immune system by sequentially switching between members of the *var* multi-gene family which encode the immunodominant surface proteins PfEMP1. However, some mechanism must exist to prevent rapid exposure of the pathogen's entire antigenic repertoire as this would quickly terminate the infection. It has previously been shown that the immune system can play an important role in orchestrating the sequential display of variants once an infection is established; however this does not explain how repertoire exhaustion is avoided in the initial phases of infection before an immune response has been established. Here we show that *P. falciparum* has evolved a highly structured switching pattern to prevent repertoire exhaustion in the early stages of infection without compromising the ability to establish new infections among partially immune individuals.

## Introduction

During blood-stage of infection with *P. falciparum*, members of the *var* gene encoded Erythrocyte Membrane Protein 1 (PfEMP1) family are exposed on the surface of infected red blood cells. Here they act as important virulence factors by mediating adherence to a variety of host cell types, causing sequestration of infected red cells in the deep vasculature [1], [2], [3], [4], [5]. PfEMP1 are also an important target for host protective antibody responses and contribute to the development of acquired immunity [6], [7]. This family of proteins has therefore been the focus of intense interest because of the role that it plays in both pathogenesis and the development of protection against clinical disease.

Mutually exclusive transcriptional switching occurs between individual members of the ∼60 *var* genes that encode this family. This changes the PfEMP1 presented on the red cell surface [8], [9], [10], resulting in an evasion of the antibody response through a process of antigenic variation [11]. To date, switching is known to be under epigenetic control, with the transcribed gene located at a specific region of euchromatin found at the nuclear periphery, [12], [13]. Silencing of the non-transcribed genes seems to involve elements in the intron and the upstream regulatory region [14] and may require the pairing of two promoters [15], [16]. Confirmation that *var* genes are expressed in a mutually exclusive manner has been obtained by the demonstration that the placing of a *var* gene promoter upstream of a selectable marker results in the silencing of the entire *var* repertoire once the marker is selected for [17], [18]. In addition to the control of the activation/repression of members of the *var* gene family, a mechanism must also exist whereby a molecular memory of the gene that was active in the previous cycle can be passed on to daughter parasites during cell division. Recent evidence suggests that one component of this memory is the selective modification of histones. Silent genes are characterised by a specific methylation of histone H3, H3K9me3, [19], [20], whereas active *var* genes are associated with the presence of H3K4me2 and H3K4me3 [20]. It has also been reported that the silencing of telomeric members of the *var* gene family is accompanied by the spreading of heterochromatin involving histone hypoacetylation and PfSIR2 [21].

While our knowledge of some of the molecular mechanisms involved in the control of *var* gene expression is accumulating rapidly, we still have very little understanding of how these processes are coordinated at the whole cell and population level in a way which provides the parasite with maximum potential to evade the immune response. We have previously proposed that structuring of parasite populations such that individual variants are only expressed one at a time might be achieved by short-lived cross-reacting antibody responses against epitopes shared between subsets of individual variants [22]. However, early infection kinetics will not be affected by these adaptive immune responses and some additional, intrinsic control might therefore be require at this stage.

Previous experiments in our laboratory have suggested that the rate at which individual *var* genes become transcriptionally activated or silenced are characteristic of that gene and relatively stable over time [23]. Recently, Frank and colleagues [24] have suggested that *var* genes that are within internal chromosome clusters have intrinsically slow off-rates whilst those in the sub-telomeres have rapid off-rates. Thus, they observe that central *var* genes tend to be the most predominantly expressed in parasites that are cultured for an extended period.

To investigate further the overall control of *var* gene expression we have derived a number of parasite clones from both the IT and 3D7 lineages and monitored *var* gene expression over an extended period of *in vitro* culture. Analysing the resulting transcription timecourses for their underlying switching dynamics we find a conserved and highly structured pattern of transcriptional change which is common to most of the clones. In an independent analysis based on optimal fitness we show how this particular pattern could have evolved as an optimal strategy between repertoire protection and immune evasion and how it allows the pathogen to successfully establish infections in non-naïve individuals.

## Results

### *Var* gene transcription profiles

We derived a number of clones from two different genotypes (IT and 3D7) and from these clones, selected a number of parasites that expressed a single dominant *var* transcript (as evidenced by Northern blot, data not shown). Using quantitative real time PCR, we then measured the expression levels of all *var* genes at various time points over an extended period of *in vitro* culture. In the resulting timecourses, transcription profiles of the initial state, as expected, were characterised by a dominant transcript with some minor transcripts also present. We chose transcription profiles of seven clones, for further analysis. Note, for simplicity, in the main figures we only present data for the five most prominent transcripts. An example showing all *var* gene transcripts of a replicate timeseries of clone 3D7_AS2 both as percentage of total signal and relative transcript level, including the experimental variation between runs, can be found in the supplementary material (Figs. S1A and B); the reproducibility of our data is further evidenced in Fig. S2 where we show the variation in transcript distribution of repeated timecourses of a single clone.

**Figure 1:**
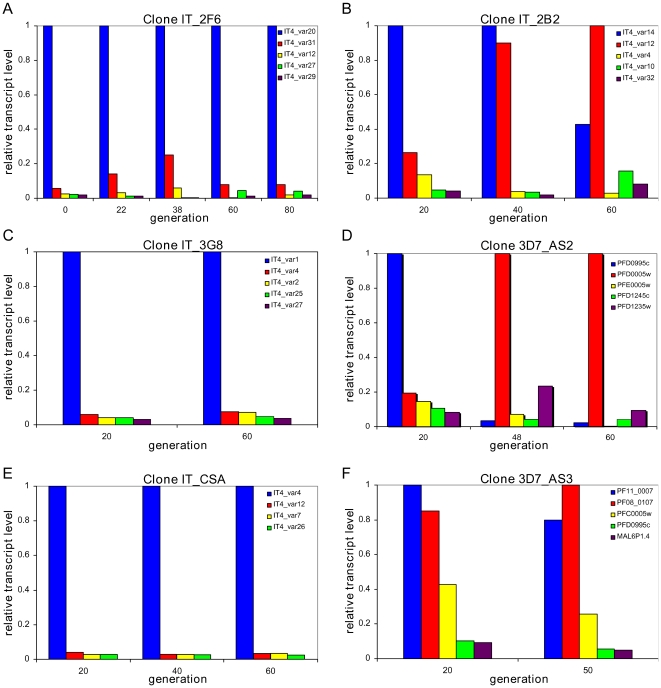
Two patterns of *var* gene activation. Parasites were cloned by limiting dilution and grown in continuous culture. From twenty generations post-cloning the expressed *var* gene repertoire of each clone was measured by quantitative PCR every few generations. Over time the clones show different reproducible patterns of *var* transcriptional change. The left panel (A, C, E) shows the clones with stable transcriptional hierarchies, while the right panel (B, D, F) shows the clones where we observed a change in the dominant transcript where a second variant replaces the initial dominant variant after 40–50 generations.

In three clones (IT_2F6, IT_3G8 and IT_CSA) we observed no change in the initial dominant transcript, which persisted for as long as we followed the culture (up to 80 generations) with small variations in the abundance of the minor transcripts (Fig. 1A, 1C and 1E and Table 1). In three other clones (IT_2B2, 3D7_AS2 and 3D7_AS3) by contrast, the initial dominant transcript declined with time and was eventually replaced by an alternate dominant transcript (Fig. 1B, 1D, and 1F and Table 1). The final clone (NF54_NR13) showed a behaviour that was intermediate between these two states. In this case, the original transcript continues to be the most abundant over 90 cycles, but other transcripts rise to levels of around 80% of the original (Fig. S3 and Table 1). The fact that we consistently find only two major types of transcriptional change in different clones strongly suggests that these are not simply down to random fluctuations or experimental oddities but must represent some inherent characteristic of *var* gene switching.

**Table 1:** Summary of analysed clones.

Parasite ID	Dominant Transcript	Genomic location, Chromosome, Promoter type
**Parasites with stable ** ***var*** ** expression**		
IT_2F6	IT4_var20	C, 7, UpsBC
IT_3G8	IT4_var1	C, 5, UpsC
IT_CSA	IT4_var4	T, 12, UpsD
NR13	PFD0020c	T, 4, UpsA
D_NF54_C3	PFD1005c	C,4, UpsB
**Parasites with switching ** ***var*** ** expression**		
3D7_AS2	PFD0995c	C, 4, UpsC
IT_2B2	IT4_var12	T, 13, UpsB
3D7_AS3	PF08_0107	C, 8, UpsC
D_NF54_C2	PF10_0406	T, 12, UpsB
D_NF54_B12E3	PFB1055c, Transfectant	T, 2, UpsB
D_NF54_B15	PFL0020w, Transfectant	T, 12, UpsB

We [23] and others [24] have previously noted that some *var* genes appear to have very slow off-rates based on stable, dominant transcription levels over many generations of *in vitro* culture; for these we would not expect to see major changes in transcript levels over the time course of the experiment. For those variants with significantly faster off-rates, on the other hand, we would expect that the culture eventually expresses a wide range of different genes and that the amount of each variant being determined by its intrinsic on- and off-rates. Instead, we observe a replacement of the dominant transcript over a timescale that is inconsistent with the idea that it is a result of direct switching between the two.

### Pattern of transcriptional change

**Figure 2:**
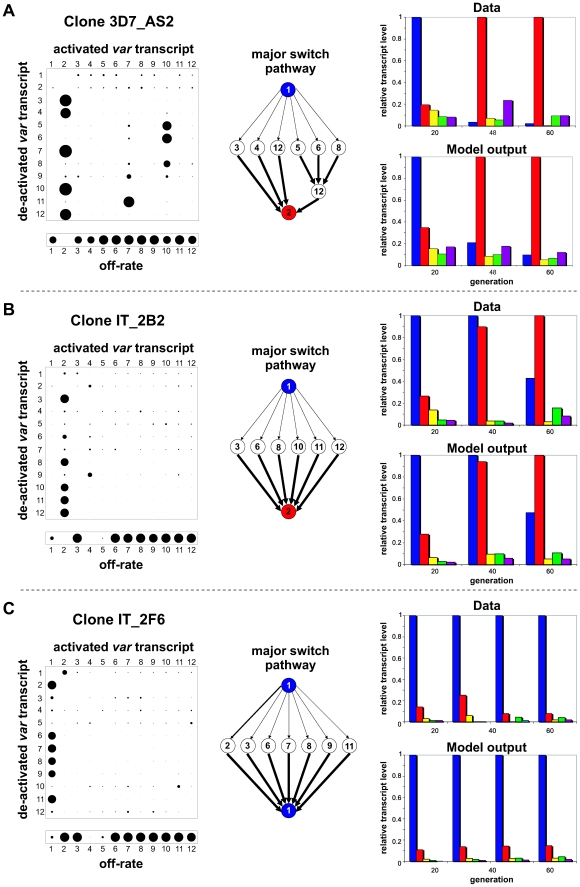
Underlying pathways of *var* gene switching. Shown are the results of our analysis of three different clones that exhibited either replacement of the dominant transcript over time, 3D7_AS2 (A) and IT_2B2 (B), or stable expression, IT_2F6 (C). The switch matrix in the left panel represents the switch biases, *β_ij_*, where the size of each circle corresponds to the transition probabilities from gene *i* to gene *j* (with 0<*β_ij_*<1); similarly for the vector below the matrix where the size corresponds to the off-rate of each individual *var* gene, *ω_i_* (with 0<*ω_i_*<0.06). In each matrix we can identify a set of genes with a high transcription bias towards the same gene (here variant 2). The switch pathway suggested by the matrix is illustrated in the middle panel where the arrows represent the switch bias (thicker arrows correspond to higher bias). On the right panel the model output for these ‘best fit’ on- and off-rates is compared to the measured transcription profiles.

To investigate this apparent phenomenon of transcript replacement more closely, we analysed the timecourses mathematically for their underlying switching dynamics. Initial studies showed that simple variation in variant growth rates could not give rise to the observed pattern (data not shown) Thus, assuming no *in vitro* growth rate differences between parasites expressing different *var* genes, the dynamics of a variant can then be described purely by its intrinsic on- and off-rates. A variant's on-rate is effectively the result of other genes switching towards this particular variant at a certain rate and bias. Bias in this context simply refers to the probability of a switch from variant *i* to variant *j*. We used an iterative process (see Methods) to find the combination of off-rates and switch biases that would best explain the observed switching pattern. In this model constraints are imposed such that *we* assume that switch rates are constant over time and necessarily require that the total sum of the switch biases of each variant add up to one. Despite the remaining large parameter space of possible on- and off-rate combinations, our method consistently converged upon a particular qualitative structure where the initial variant switches at medium off-rate with no preferential bias to a subset of variants. Each variant in this subset has a high off-rate and a high transcription probability biased towards a single new variant. We refer to this structure hereafter, for simplicity, as the single-many-single or *sms* pathway. Fig. 2 shows the result of our analysis for three data sets. The left panel depicts the resulting switch-matrices, where the size of each circle in row *i* and column *j* corresponds to the transcription probability from variant *i* to *j*, and the off-rate vectors where the size of each circle corresponds to the variant's off-rate. Note, in our analysis we only used a subset of the *var* transcripts, in this case the 12 most dominant variants, which we determined to be optimal given the available data (see Methods). Most other *var* gene transcripts remain at very low levels over the entire time course (see e.g. Figs. S1 and S2), however, and these are unlikely to have a significant effect on the observed switching pattern.

The predicted *sms* pathway can be seen within the matrix as an unbiased switch away from the initial variant (here variant 1) to a set of variants with a strong bias towards the second dominant variant (here variant 2). This particular pattern is illustrated by highlighting the major switch pathways as flow-diagrams in the middle panel of Fig. 2. In every case the initial variant switches to a group of variants which then switch at high rate and similar bias to another variant that will then become the dominant transcript. The right panel shows the qualitative comparison between the experimental transcription profiles (of the five most prominent transcripts) and the timecourses generated by our model. In each case there is good agreement between the data and model output. In line with Fig. 1 and for illustrative purposes only we chose to show only a subset of variants; an example showing all 12 variants can be found in the supplementary material (Fig. S4A and B). To compare the fit of the predicted switch pathway to other possible pathways we applied various constraints to our model such that only one or a small number of variants are allowed to have switch biases and thus contribute to the observed switching pattern (see supplementary Fig. S5). This clearly showed that simple differences in switch rates could not explain the data. It also highlighted the fact that a direct one-to-one switch from the first dominant variant to the second one is incompatible with the observed data.

We also considered the parasite clones in which a single dominant transcript in these profiles remained stable. One feature of these data that was difficult to explain was the fact that a series of minor transcripts was always present and in most cases their abundance showed slight or significant fluctuations over time. Since all of these parasites are clonal, the minor transcripts must have arisen from some daughters switching away from the original *var* type present in the original clone. Why then did this switching process not continue so that the proportion of the dominant transcript decreased observably over time? Applying the above analysis to this series of data we discovered that in these cases also, an SMS pathway was the best fit to the data, as exemplified by Fig. 2C. Our analysis thus suggests that although these clones exhibit a phenotype of stable expression of a single variant, a much more dynamic situation may exist in which the dominant *var* gene is continuously switching to a subset of other *var* genes (the minor transcripts that fluctuate) which continue to switch back to the original dominant transcript.

Finally we applied our analysis to transcription profiles previously generated by Frank *et al.* [19] and found the same switching pattern underlying their data (see Fig. S6). The fact that we can recapture the same pattern from two independently derived sets of data from different parasite genotypes and multiple independent clones strongly suggests that this particular pathway is an intrinsic feature of *var* gene switching.

### Optimal switching pattern

We next investigated why this unusual pattern of switching might have evolved. *In vivo*, *P. falciparum* is faced with two opposing pressures. If the host is rapidly exposed to the majority of the antigenic repertoire, then there is the danger of the elimination of the parasite by the immune response. Thus the parasite needs to minimise the proportion of the antigenic repertoire to which the host will become exposed. At the same time, in order to maximise the potential for immune evasion, every *var* gene should be readily accessible, in terms of being switched to, from every other gene within the repertoire. To investigate if the observed pathway could have arisen as a result of this evolutionary conflict, we envisaged the *var* gene repertoire as a network in which the nodes represent individual gene variants and the edges the transition, i.e. switches, between them. We used a genetic algorithm to ‘evolve’ an initially random network to optimise over two traits: (i) average distance through the network, which corresponds to repertoire protection and indirectly infection length, and (ii) robustness to the removal of individual nodes, which corresponds to the ability to adapt to selection pressure, e.g. through pre-existing antibody responses.

**Figure 3:**
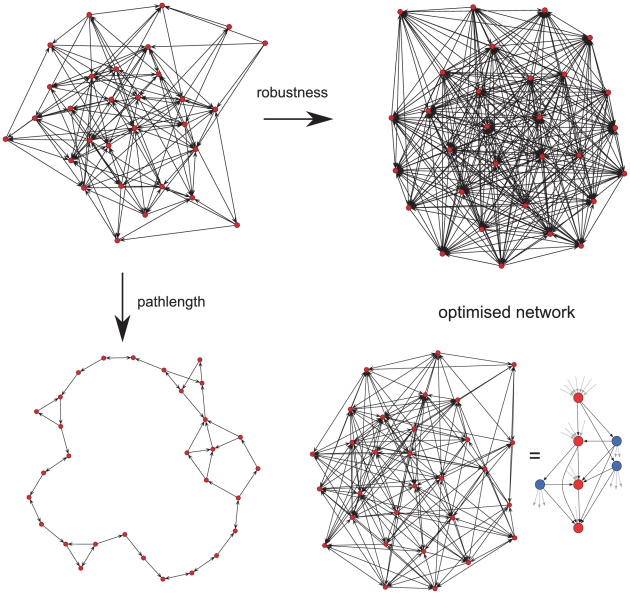
Network optimisation over two evolutionary traits. An initially random network (top left) can be evolved to optimise either robustness (top right), i.e. the potential for evading immune responses, resulting in a fully connected network, or path length (bottom left) which minimised repertoire exposure to the immune system and thus results in a ring-like structure with minimum connectivity between nodes. Optimising over both traits simultaneous produces a network consisting of variants which either switch to many other variants or being switched to from many other variants (bottom right). The lattice-like structure is highlighted to the right of the network, indicating ‘sink’ (red) and ‘source’ nodes (blue).

As expected, optimising a network to maximise robustness led to a fully connected network where variants switch to every other variant within the network, whereas optimising for repertoire protection alone results in a ring-like structure where every variant switches to one other variant only (Fig. 3). Optimising over both traits simultaneously, however, results in a lattice-type network containing nodes with either a high out-degree, i.e. variants that switch to a high number of other variants, or nodes with a high in-degree, i.e. variants which are being switched to by a high number of other variants. Together, these ‘source’ and ‘sink’ nodes, highlighted in blue and red in Fig. 3, respectively, form the basis of an expansion - contraction process that embodies the evolutionary trade-off between adaptability and repertoire protection in *var* gene switching.

We note that this expansion – contraction process incorporated in the ‘*lattice*-type’ switching pattern closely resembles the *sms* pathway we predicted to underlie the observed *in vitro* switching. However, the optimised network does not take into account switch rates and biases but rather presents a net flow, or transition, between any two variants. For a better qualitative comparison we can represent the switching matrices together with their respective off-rate vectors as a directed network where each edge corresponds to the switch direction from one variant (node) to another, simply calculated from the sign of the net transition, 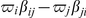 (see Methods). In this case we find the resulting network again divided into nodes with either a high in-degree or out-degree, shown in Figs. S7A and S7B for clones 3D7_AS2 and IT_2B2, respectively, underlining the similarity between the *sms* and *lattice*-type pattern.

#### (i) Comparison of switching pathways during primary infections

**Figure 4:**
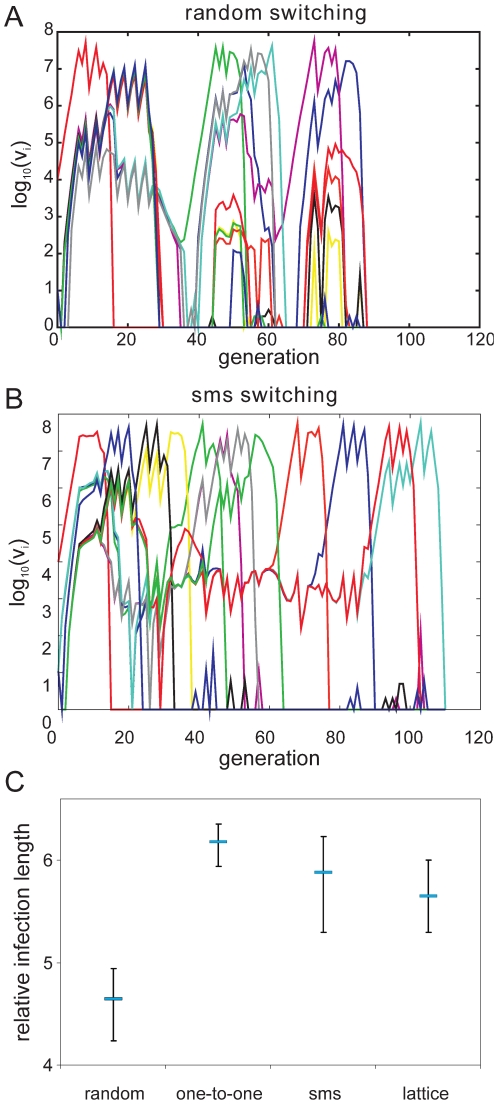
Effects of switching pattern on malaria infection dynamics. Simulating malaria infections in the naïve host, here shown as parasitaemia levels of the various antigenic variants under two different assumptions about the nature of switching, does not reveal major qualitative differences between random and preferential switching (A and B, respectively). However, a marked increase in infection length, as measured in multiples of a single variant infection, can be observed once switching is more structured (C). Shown are the median (blue bars) and lower and upper quartiles of 500 model realisations.

To investigate how a pre-determined switching pathway might affect the *in vivo* dynamics of parasite growth, we simulated various switching patterns during the early phases of malaria infection in a naïve individual by means of a simple within-host model, the details of which we have previously published ([22], plus see Methods and supplementary material). We considered four different switch pathways: (i) *random*, with no inherent switch biases, (ii) a simple *one-to-one* switch where each variant predominantly switches to one other variant, and the two highly structured switch pathways predicted by the network optimisation and data analysis, (iii) *lattice*-type and (iv) *sms*-type switching, respectively. While we found no discernible differences in the ability to establish an infection (Fig. 4A and 4B), we observed an increase in the duration of infection as switching becomes more structured (Fig 4C). To obtain results that are independent of particular parameter values, especially those concerning the immune response, we measured Infection length in this context as multiples of a single variant infection.

#### (ii) Effect of switching pathways during subsequent infections

We then examined the effect of structured switching in individuals with pre-existing immunity to a number of antigenic variants. We simulated re-infection by ‘clearing’ an ongoing infection and re-challenging the host with the same pathogen, i.e. with the same antigenic repertoire and switch pathway. This is of particular interest as it takes into account the actual order at which variants appear during the initial stages of infection as dictated by both the switch pathway and the antigenic relationship between the variants. As each variant triggers both long-lived variant-specific and temporary cross-reactive responses (see full model details in the supplementary material) we made sure to leave enough time between clearance and re-challenge to allow the short-lived responses to decay.

**Figure 5:**
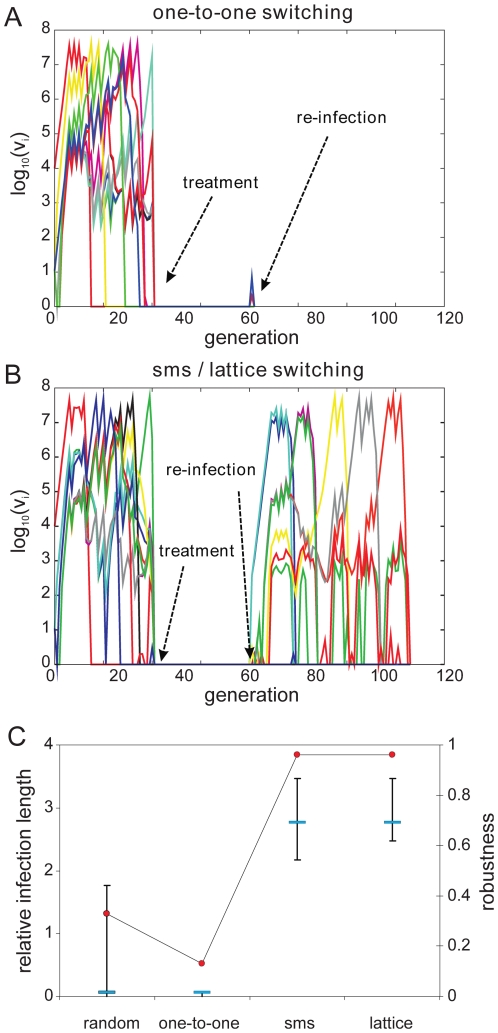
Effects of switching pattern on malaria infection dynamics during re-infection. Simulating infection, clearance and re-infection by the same pathogen reveals the vulnerability of the highly ordered one-to-one switching pathway (A). The expansion-contraction process within the *sms* pathway allows for greater flexibility to overcome the inhibitory responses to find an alternative route of expression (B). This is clearly demonstrated by both robustness, measured as the proportion of runs where secondary infections were successfully established, and the length of secondary infection (C). Shown are the median (blue bars) and lower and upper quartiles of 500 model realisations.

As expected, pre-existing immune responses greatly reduce the parasite's propensity to establish infections. However, we found that the more structured *sms* and *lattice*-type switch pathways are far more efficient at establishing a secondary infection than either *one-to-one* or *random* switching. Fig. 5A demonstrates how the strict switch hierarchy in the *one-to-one* pathway can quickly lead to immune elimination as the pre-existing responses rapidly react to the first set of variants and clear the infection. In contrast, the expansion-contraction process embodied by both the *sms* and *lattice*-type pathways is sufficiently flexible to overcome these constraints (exemplified in Fig. 5B). Importantly also, there is no significant difference between the *sms* and *lattice*-type pathways in their ability to successfully establish a secondary infection or in the duration of the ensuing infections (Fig. 5C), reinforcing the notion that these two pathways are, at least qualitatively, equivalent.

## Discussion

Establishing chronic infections is particularly important among vector borne pathogens since vector abundance may be seasonal or otherwise uncertain. For pathogens with a limited antigenic repertoire, such as *P. falciparum*, control over variant expression is therefore essential. Despite some differences in results and interpretation, it is becoming clear that the *var* gene repertoire of *P. falciparum* is divided into slow and fast switching phenotypes (this paper, [23], [24], [25]). This could potentially introduce a switch hierarchy by which stable variants are more prominently expressed during the early phases of infection. However, with only ∼60 members of the *var* gene family among which to switch [26] and typical clinical parasite burdens of >10^10^, it is very difficult to envisage how this partitioning of on- or off-rates alone could prevent the entire repertoire from being expressed early on.

Here we report that *var* gene switching might occur in a highly structured pattern which can offer a partial solution to this problem. This particular pathway not only depends on inherent differences in the rates at which *var* genes become transcriptionally active or silent but crucially on intrinsic switch biases between individual genes. Importantly, we also found that very high on-rates and very low off-rates can both be explained by the same principal mechanism of biased switching in which a subset of variants switch at high bias either to a new variant or back to the original. We therefore note that *var* gene activation cannot be simply seen as an ‘intrinsic’ property but should be viewed in context of a whole *var* gene switching network. This further implies that the fate of a gene is crucially dependent on the ‘starting position’ within this network such that a variant that quickly gains dominance in a particular situation might not reach significant levels under different circumstances if it is part of a different ‘sub-network’, i.e. when it does not get switched to at sufficiently high rates from other variants, and *vice versa*.

Other antigenically variable organisms such as *Trypanosoma* spp or *Borrelia hermsii* also exhibit programmed sequences of gene activation [27], [28], [29], [30]. In contrast to *P. falciparum*, however, these may partly be mediated by sequence homologies between the expression site and the donor site used for recombination [31]. One major drawback of tightly ordered gene activation is that it requires every subsequent variant to be able to evade current immune responses and therefore may be compromised by previous infections. For organisms such as *T. brucei* or *B. hermsii*, which predominantly infect naive hosts or are less constrained in their generation of antigenic diversity during infection, this is not a major problem. For *P. falciparum*, however, most infections occur in non-naive individuals and complete discordance between the infecting parasite and the immune repertoire of the host cannot be guaranteed. Furthermore, the rate of mitotic recombination between *var* genes [32], [33] is unlikely to be fast enough to evade pre-existing immune responses. The initial expansion or diversification process towards a group of variants within the *sms* pathway might therefore significantly improve the chance of evading early immune responses whilst the subsequent contraction protects the remaining repertoire from further exposure.

With regards to how the aforementioned trade-off within which this particular switch pattern has evolved it is interesting to note that it represents two selective forces acting at both the within- and the between-host levels. That is, the within-host infection dynamics are dominated by the pathogen's need to survive for as long as possible to enhance its chance for onward transmission. This requirement would usually favour a tightly regulated sequence of gene activation to minimise the exposure of the parasite's antigenic repertoire. On the other hand, though, a strict order of expression together with its accompanying immune signature would leave the parasite highly vulnerable when encountering hosts with previous exposure to similar strains. Therefore, having a more flexible yet still structured switch pattern, as the one reported here, could potentially ease competition between antigenically similar strains. Furthermore, as the activation of gene variants appears to be governed by the whole *var* gene switching network, and in particular the starting variant, population level exhaustion of potentially dominant, i.e. intrinsically over-expressed variants is further minimised.

Switch or activation hierarchies have previously been proposed to explain the sequential appearance of antigenic variants during trypanosome infections [34], [35]. Although it was indicated that this coordinated expression can occur even with a small variant repertoire [35], it is unclear whether it can be stably maintained over longer periods. We have previously demonstrated that immune mediated selection, by means of short-lived cross-reacting antibody responses against shared epitopes, can structure the parasite populations into sequential dominance of individual variants [22]. While this model was very successful in producing chronic infection, the time taken to establish the cross-reactive antibody responses *in vivo* meant that the model could not accurately reflect early infection kinetics where parasite intrinsic factors, such as structured switching, are more likely to play a role. The *sms* pattern of switching reported in this paper has the potential to unite the two mechanisms by producing a realistic progression in expression of variants in the early stages of infection while setting up the conditions, in this case, a network of partially cross-reactive responses, that reliably leads to chronic infection.

The antigenic relationship between the variants within a specific switching pathway also appears to play an important role. In particular, the model predicts that in both the *sms* and *lattice*-type switching pathways the initial switch should be to a set of antigenically similar variants which then all switch to an antigenically distinct one. In this process, ‘switch intermediates’ are effectively controlled by the cross-reactive responses elicited by the initial variant and can therefore be ‘used’ again during the later stages of infection. This conclusion would be consistent with the *in vivo* observations of Kaestli *et al.* [36] that observed the reappearance of the same variant in patients monitored longitudinally.

What are the implications of our findings for the molecular mechanisms that underlie the switching process? Frank *et al.* [24] suggested from their experiments that the expression of a stable, non-switching transcript is associated with centrally positioned *var* genes (those bearing an UpsC type promoter sequence) whereas rapidly switching *var* genes are located in the sub-telomeres. We also see a preponderance of central genes in the non-switching clones but also telomeric genes such as PFD0020c and var2CSA from both genotypes. Similarly we note a 3∶1 ratio of telomeric to central genes in those clones that switched rapidly. Thus an association with genomic position may exist, but this is not absolute. In the data that we have available, we also observe that switches occur only to *var* genes located on other chromosomes, or to *var* genes located in central versus telomeric clusters on the same chromosome. Switches to closely linked genes appear to be prohibited unless accompanied by a local deletion event [37], [38]. It has been shown that active *var* loci occupy a ‘transcriptionally permissive zone’ in the parasite nucleus [39] as part of a cluster of telomere ends [33]. Therefore, it may be that other *var* genes in the cluster containing the active gene are favored for activation. We were unable to find any strict association of these switching patterns with primary sequence features. However, these data now permit a systematic description, perhaps through parasite transfection experiments, of the sequences and molecules responsible for these switching patterns.

Together, our results highlight the intriguing interplay between parasite-controlled switching and immune-mediated selection and reinforce the hypothesis that structured switching in *P. falciparum* has evolved as an evolutionary compromise between the protection of its limited antigenic repertoire and the flexibility to fully utilise this repertoire when needed.

## Methods

### Experimental procedure

Quantitative ‘real-time’ PCR was performed using a Rotorgene thermal cycler system (Corbett Research). Reactions were performed in 15 µl volumes using 2X QuantiTect SYBR Green PCR master mix (Qiagen), var-specific primers at .5 µM, and the appropriate volume of DEPC-treated H20 (Qiagen). The PCR cycling conditions were further optimized for P. falciparum cDNA were 95°C for 15 min followed by 40 cycles of 94°C for 30 s, 58°C for 25 s and 68°C for 30 s followed by a final extension step at 68°C for 10 minutes. To give more consistent reaction efficiency, we found it necessary to redesign seven primer sets which were placed near or inside the transmembrane-encoding sequence (Supplementary Methods): PFI1830c, PF08_0106, PF07_0139, PF11_0008, PFD1000c, PFD1245c, and PFD1015c. Primers were stored at a 10× concentration at 4°C and cDNA was kept in single-use aliquots. The fluorescent signal was acquired at the end of the elongation step of each reaction cycle. After the reaction, product specificity was verified by melting-curve analysis and gel electrophoresis of each PCR product.

Quantification using the ‘Comparative Quantitation’ method packaged with ROTORGENE software version 6.0. All primer pairs were tested on identical aliquots of genomic DNA, and the median ‘Take-Off Point’ value for the primer set was calculated. The ‘Take-Off Point’ is analogous to the ‘CT-value’ employed by the ΔΔCT method, except the ‘Take-Off Point’ is computationally determined and its measurement does not require a standard curve for each primer set. Furthermore the ‘Take-Off Point’ is based on the kinetics of each reaction, not a critical fluorescence value that may favour certain transcripts over others. Primer pairs with ‘Take-Off’ values varying by +/− 50% of the median value when tested on the same sample of DNA were redesigned and retested. To account for amplification bias in the reaction conditions, a correction factor equal to the average variation from the mean ‘Take-Off’ point over 5 trials was applied. We used seryl-tRNA synthetase as an endogenous control as it displayed the most uniform transcription profile in different parasite isolates and an unchanged pattern throughout the parasite life cycle. All transcript levels were then normalised with respect to the most abundant variants as this allowed for better comparison in transcript levels and their respective change over the time course.

### Analysis of transcription profiles

We devised a time-discrete model to describe the change in the proportion of *var* gene transcripts from generation to generation, assuming each variant has a constant rate and bias at which it will switch towards another variant. The proportion of variant *i*, *v_i_*, at generation *t*+1 is therefore the sum of variants *j* switching towards variant *i* minus the proportion that has switched away from variant *i*. The dynamics of the variants can then be written as follows:
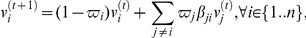
with *v_i_(t)* =  proportion of variant *i* at generation *t, ω_i_* =  off-rate of variant *i*, and *β_ji_* =  switch bias from variant *j* to variant *i*.

To determine the switch matrix, (*β_ji_*), and off-rate vector, (*ω_i_*), we used a Markov Chain Monte Carlo (MCMC)-like method to find the best model fit to the data by iteratively modifying the switch rates and switch biases. An initial matrix and off-rate vector are randomly filled and then repeatedly subjected to small perturbations. At each iterative step, i.e. after each perturbation, we calculated the deviation between data and model output by defining the following error:
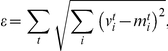
where 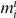 is the measured transcript level of variant *i* at time point *t* and 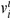 is the model output. If the perturbed matrix and vector yield a smaller error than the original ones they will be updated and again subjected to small perturbations. This process is repeated until a chosen convergence criteria (on ε) is fulfilled.

Because of the high number of free parameters and small number of available data points we chose to use a reduced system. That is, instead of trying to fit the full 60×60 switch matrix and 60 off-rates we used a 12 dimensional matrix and vector instead. This was also motivated by the fact that only a subset of measured transcript was above a 5% confidence level. However, we also investigated smaller and bigger systems and found that while this did not change the qualitative nature of the results presented here, the 12 dimensional system seemed optimal in terms of computational speed, goodness-of-fit and convergence. That is, using a much reduced system resulted in a noticeably poorer fit whereas increasing its dimension did not significantly improve the fit between model outcome and the data after a given number of iterations (see Fig. S8).

### Genetic algorithm

To determine an optimal switch strategy between immune evasion and repertoire protection we employed a genetic algorithm. The aim was to optimise a network for both a) average distance through the network (corresponding to infection length), and b) robustness to the removal of nodes (corresponding to evading ongoing or pre-existing immune responses). Average distance was defined as the mean number of edges that must be traversed by the shortest path between every pair of nodes in the network (the geodesic distance), normalised to a value between zero and one by dividing by the maximum possible. Robustness was measured as the average proportion of nodes that must be removed in order to fragment the network into more than one component, based on 500 simulations of the progressive removal of random nodes for each network. A simple multiplicative fitness function was defined based on these network parameters, since both were normalised to values between 0 and 1, and randomly generated networks were modified iteratively; random deletions and additions of edges that improved the network's fitness were kept and built upon, whereas random deletions and additions that lowered its fitness were discarded.

### Infection model

To simulate the effect of structured switching on malaria infection dynamics we employed a stochastic, mathematical model based on a previous antigenic variation framework [20]; full model details can be found as online supplemental content (Text S1).

## Supporting Information

Figure S1 Replicate timecourse of clone 3D7_AS2. Transcription levels of all 60 var genes as percentage of the total signal (A) and relative to the dominant var transcript (B) at generations 20 and 60 post-cloning. Shown are the averages of two duplicates with the error-bars indicating the variation between experiments.
(1.05 MB TIF)

Figure S2 Replicate transcript levels of a stable clone, 3D7_AS6. Shown are the average transcription profiles of clone 3D7_AS6 and six sub-clones, measured at 20 generations post cloning, clearly demonstrating the reproducibility of our data and relatively low between-experiment variations. The standard deviations are shown as error bars.
(0.64 MB TIF)

Figure S3 Transcription timecourse of clone NF54_NR13. Detailed timecourse of the transcription levels of the five most abundant var gene transcripts. The switch pattern appears as a mixture between the behaviour of stable and unstable clones with the initially dominant variant remaining dominant over the whole time course while other variants displaying a more dynamic state.
(0.55 MB TIF)

Figure S4 Transcript level time course of clone 3D7_AS2. Shown are the 12 most dominant var gene transcripts from clone 3D7_AS2 (figure 1D, main text) used for the iterative method after 20 (black bars), 48 (white bars) and 60 (grey bars) generations post cloning (A) and in comparison the model output of the same 12 variants (B).
(0.44 MB TIF)

Figure S5 Testing the model under various constraints. To compare the model fit to other possible switching scenarios we applied a number of constraints to our model and then tried to optimise under these constraints. It is clear that neither simple differences in off-rates (A) nor a simple one-to-one switch (B) can explain the data. By allowing more variants to be part of the switch pathway, (C) and (D), the method immediately converges towards the sms-type switching, although not all variants will be part of this primary pathway (D).
(1.03 MB TIF)

Figure S6 Predicted switching pathways of switching clones. Shown are the data and simulation results for a series of switching clones, D_B12 (A) and D_C2 (B), described by Frank et al. (2007). The switch matrices in the left panels represent the switch biases, β_ij_, where the size of each circle corresponds to the transition probabilities from gene i to gene j; similarly for the vector below the matrix where the size corresponds to the off-rate of each individual var gene, ω_i_. The switch pathway predicted by our model (middle panel) is in agreement to the sms pathway found in our data. The right panels compare the model output for these ‘best fit’ on- and off-rates to the experimental data.
(0.99 MB TIF)

Figure S7 Network representation of in vitro transcription pathways. The predicted networks describing transcriptional change in clones 3D7_AS2 (A) and IT_2B2 (B) consist of either source (blue) and sink variants (red) and are similar to the one predicted through the network optimisation.
(1.92 MB TIF)

Figure S8 Model output in dependence on parameter space. Throughout our analysis we used a reduced system of 12 variants. Given the available data this seemed a good compromise between goodness-of-fit and statistical and computational feasibility. Using a bigger parameter space of 20 variants (A) does result in a slightly improved fit to the transcription data of clone 3D7_AS2, compared to 12 variants (B), whereas a much further reduced system leads to a noticeably less good fit (C). Importantly, in all cases the qualitative switch pathway remains mostly invariant and predicts an initial switch to a number of intermediates and then towards the second dominant variant (which can be seen as significant column biases towards the second variant). Note, as the value of ε is dependent on the dimension of the analysed system we cannot make a direct quantitative comparison between the three models.
(0.78 MB TIF)

Text S1 Detailed description of the stochastic within-host model.
(0.07 MB PDF)
