# Supplementary figures and images for: Antigenic Variation in Plasmodium falciparum Malaria Involves a Highly Structured Switching Pattern
Source: PLoS Pathog. 2011 Mar 3;7(3):e1001306. doi: 10.1371/journal.ppat.1001306 (PMC3048365; doi:10.1371/journal.ppat.1001306)

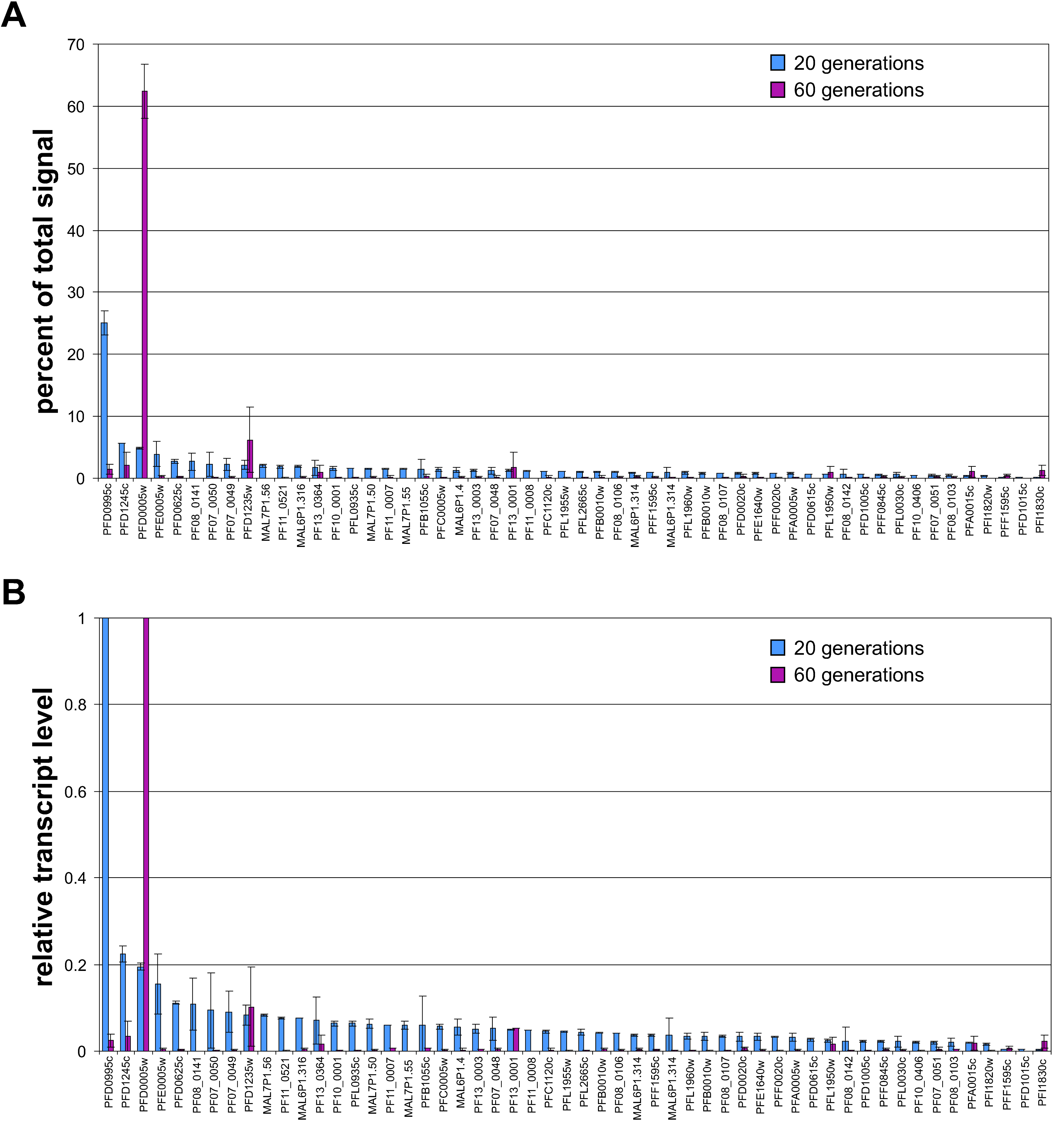

Supplement: Figure S1 — Replicate timecourse of clone 3D7_AS2. Transcription levels of all 60 var genes as percentage of the total signal (A) and relative to the dominant var transcript (B) at generations 20 and 60 post-cloning. Shown are the averages of two duplicates with the error-bars indicating the variation between experiments. (1.05 MB TIF) [file ppat.1001306.s001.tif]

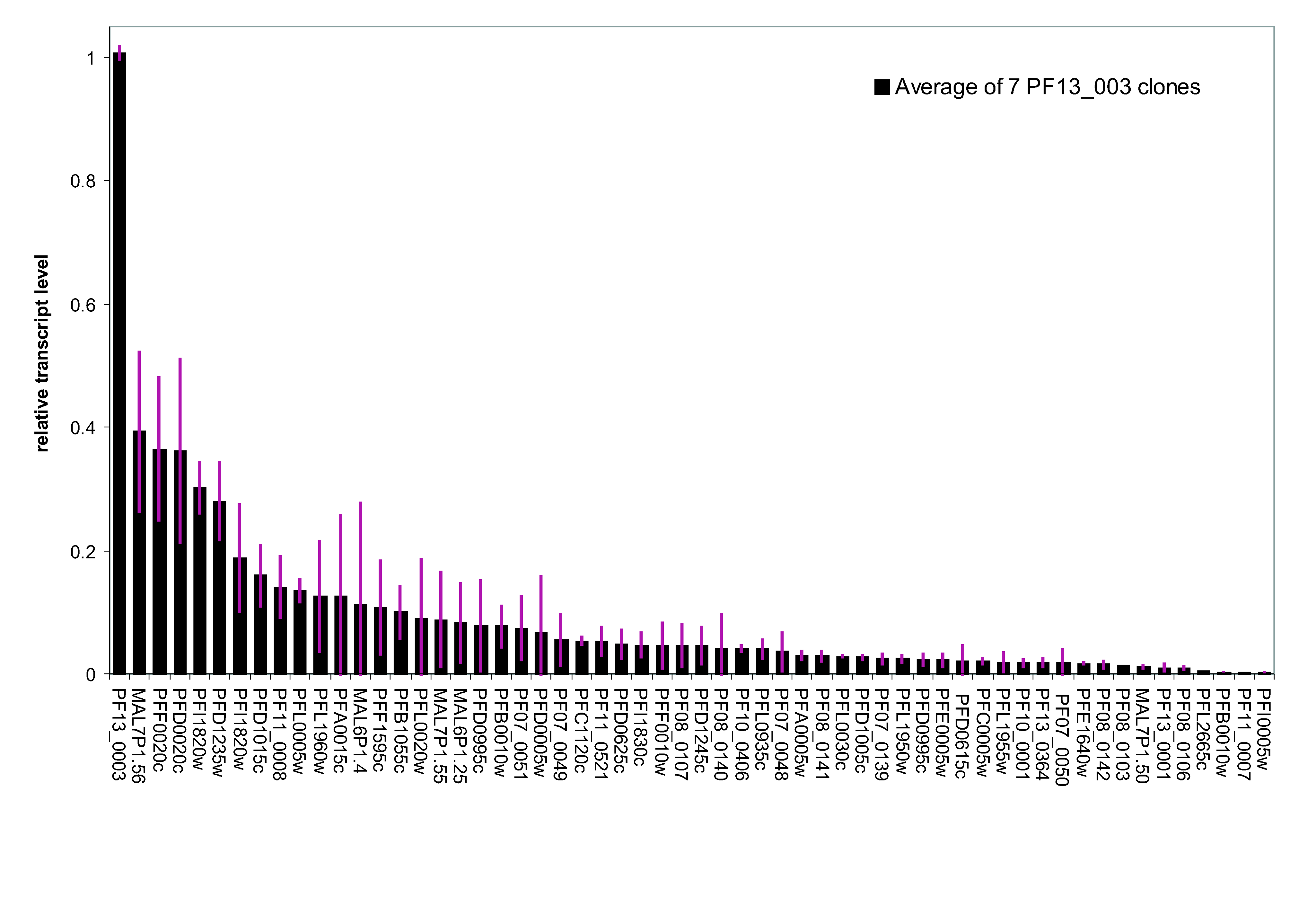

Supplement: Figure S2 — Replicate transcript levels of a stable clone, 3D7_AS6. Shown are the average transcription profiles of clone 3D7_AS6 and six sub-clones, measured at 20 generations post cloning, clearly demonstrating the reproducibility of our data and relatively low between-experiment variations. The standard deviations are shown as error bars. (0.64 MB TIF) [file ppat.1001306.s002.tif]

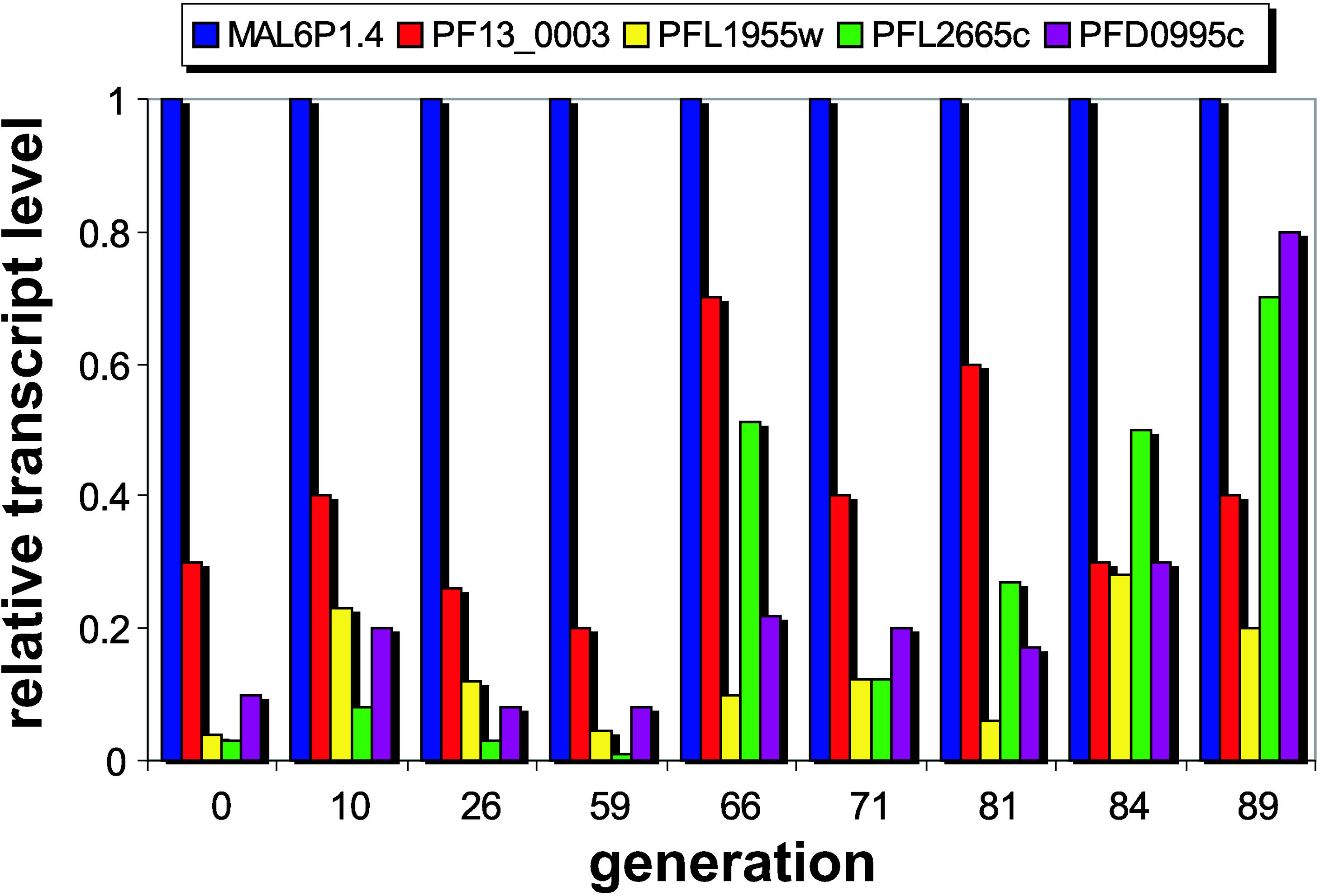

Supplement: Figure S3 — Transcription timecourse of clone NF54_NR13. Detailed timecourse of the transcription levels of the five most abundant var gene transcripts. The switch pattern appears as a mixture between the behaviour of stable and unstable clones with the initially dominant variant remaining dominant over the whole time course while other variants displaying a more dynamic state. (0.55 MB TIF) [file ppat.1001306.s003.tif]

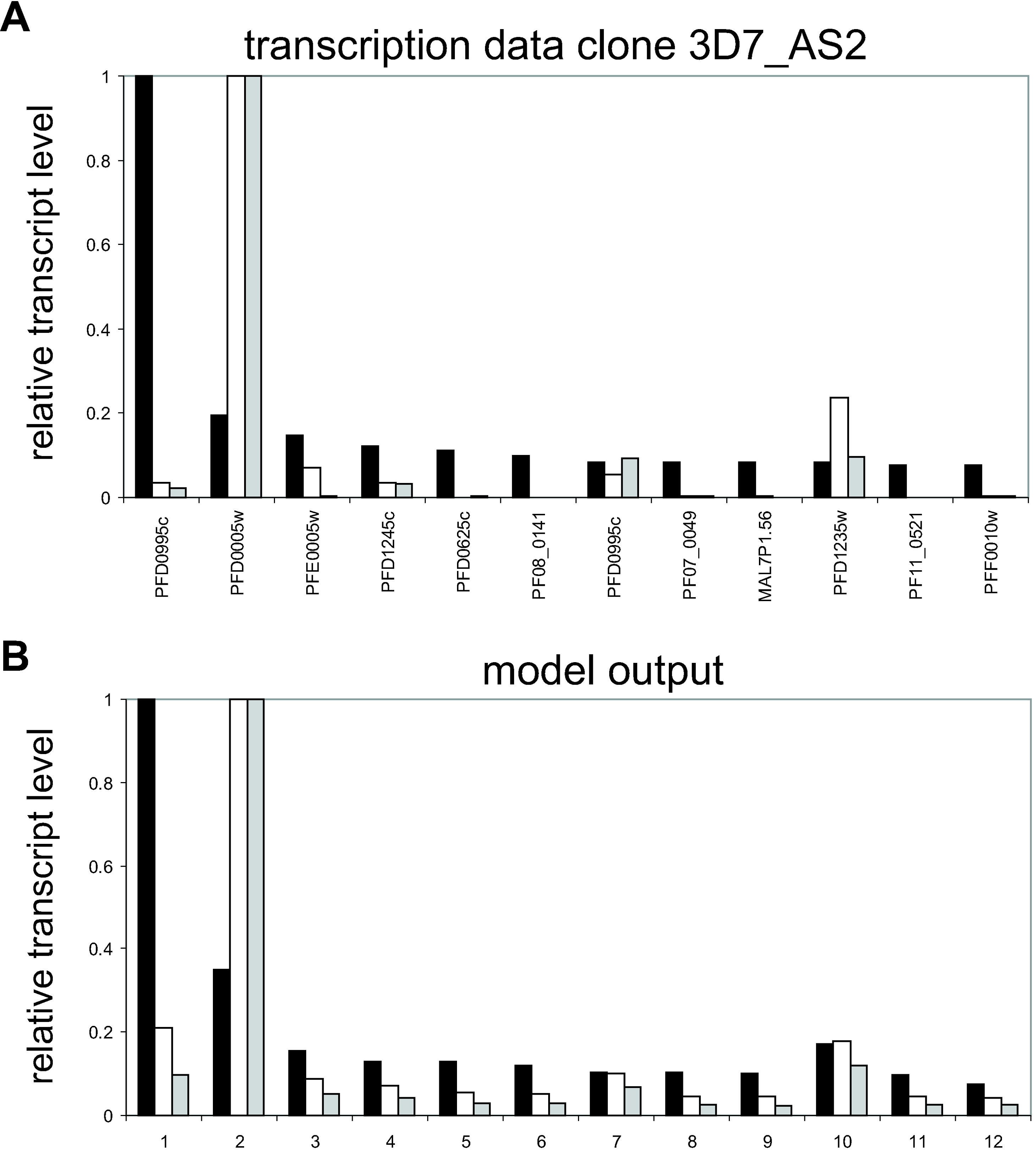

Supplement: Figure S4 — Transcript level time course of clone 3D7_AS2. Shown are the 12 most dominant var gene transcripts from clone 3D7_AS2 (figure 1D, main text) used for the iterative method after 20 (black bars), 48 (white bars) and 60 (grey bars) generations post cloning (A) and in comparison the model output of the same 12 variants (B). (0.44 MB TIF) [file ppat.1001306.s004.tif]

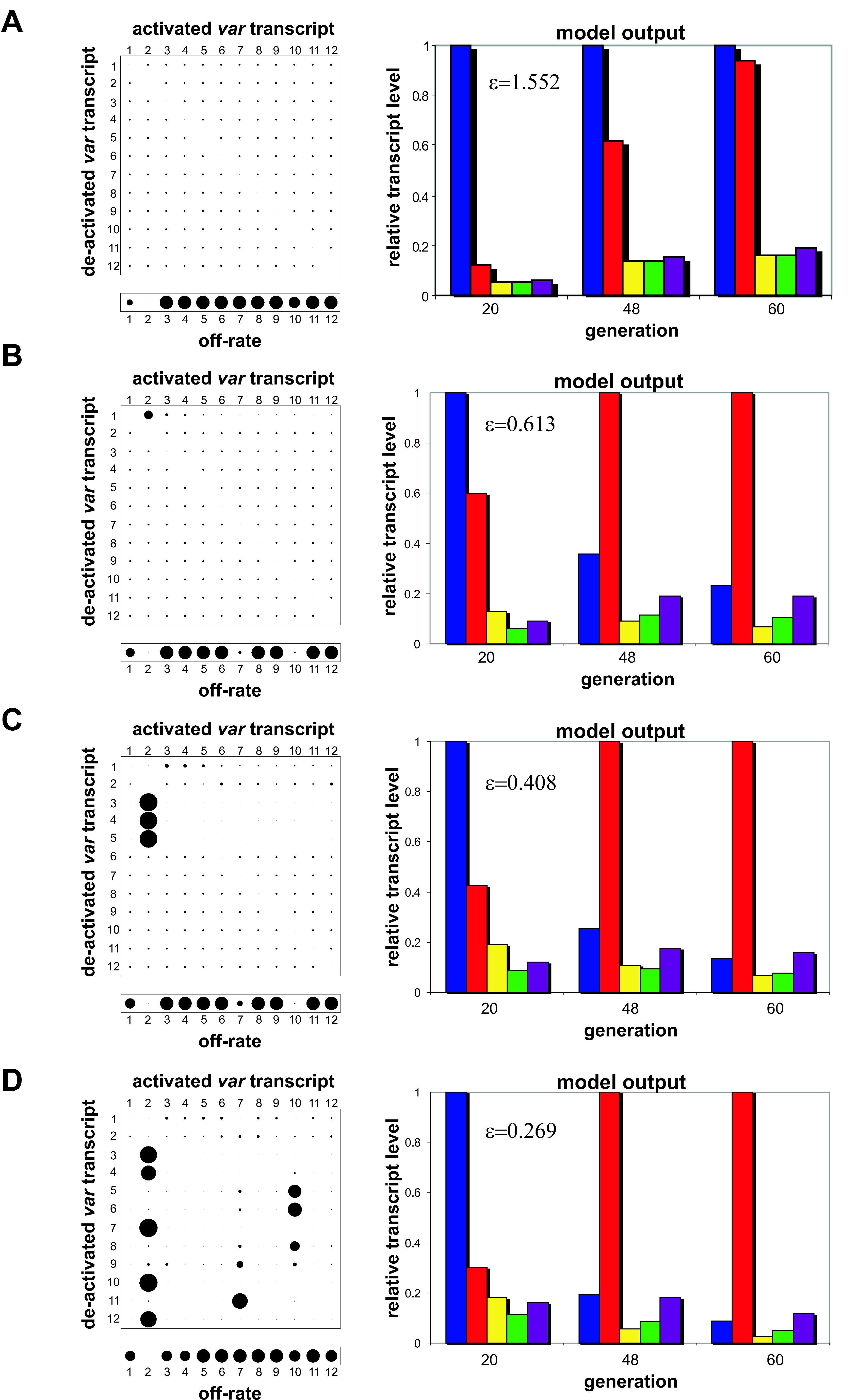

Supplement: Figure S5 — Testing the model under various constraints. To compare the model fit to other possible switching scenarios we applied a number of constraints to our model and then tried to optimise under these constraints. It is clear that neither simple differences in off-rates (A) nor a simple one-to-one switch (B) can explain the data. By allowing more variants to be part of the switch pathway, (C) and (D), the method immediately converges towards the sms-type switching, although not all variants will be part of this primary pathway (D). (1.03 MB TIF) [file ppat.1001306.s005.tif]

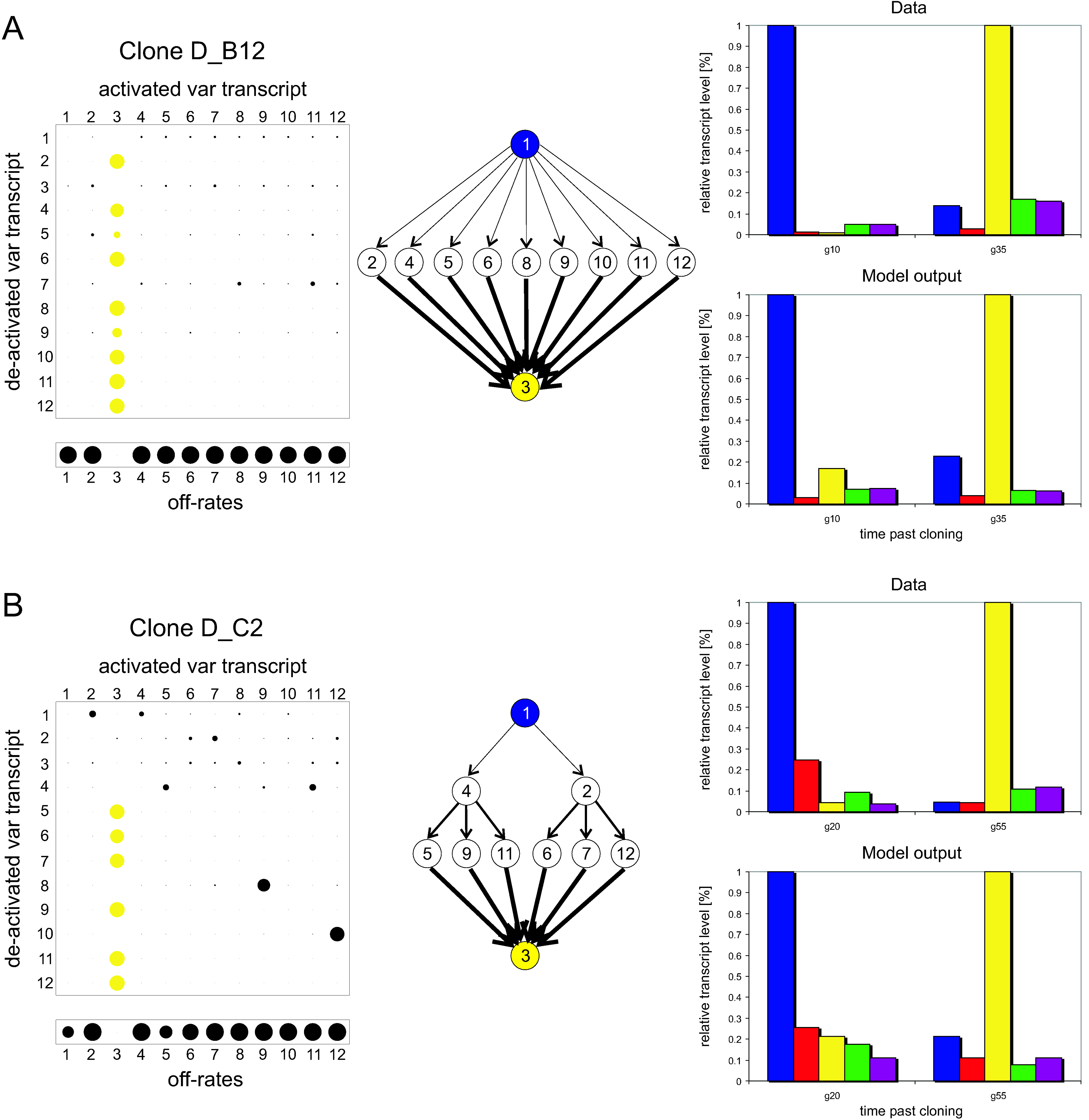

Supplement: Figure S6 — Predicted switching pathways of switching clones. Shown are the data and simulation results for a series of switching clones, D_B12 (A) and D_C2 (B), described by Frank et al. (2007). The switch matrices in the left panels represent the switch biases, βij, where the size of each circle corresponds to the transition probabilities from gene i to gene j; similarly for the vector below the matrix where the size corresponds to the off-rate of each individual var gene, ωi. The switch pathway predicted by our model (middle panel) is in agreement to the sms pathway found in our data. The right panels compare the model output for these ‘best fit’ on- and off-rates to the experimental data. (0.99 MB TIF) [file ppat.1001306.s006.tif]

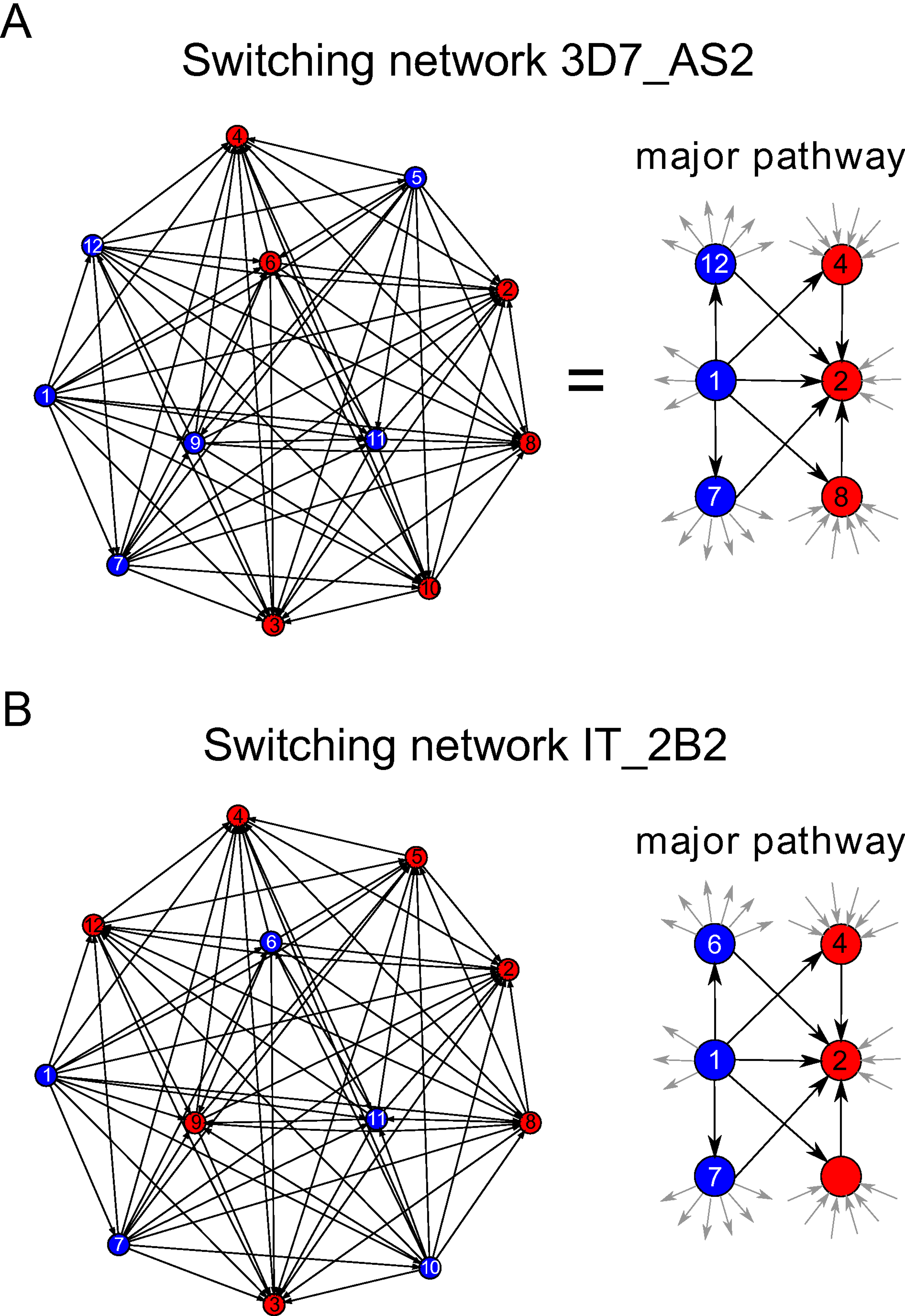

Supplement: Figure S7 — Network representation of in vitro transcription pathways. The predicted networks describing transcriptional change in clones 3D7_AS2 (A) and IT_2B2 (B) consist of either source (blue) and sink variants (red) and are similar to the one predicted through the network optimisation. (1.92 MB TIF) [file ppat.1001306.s007.tif]

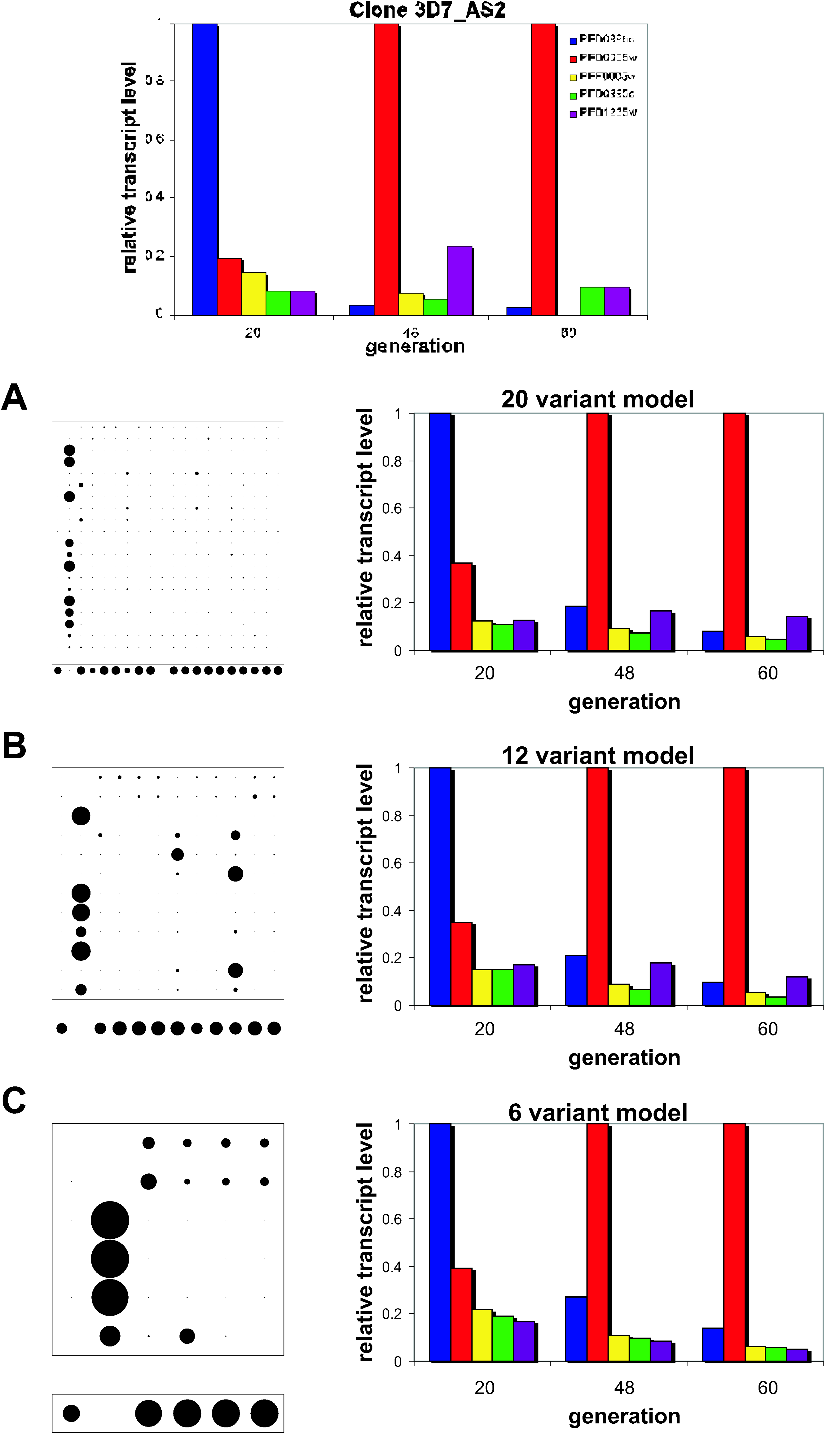

Supplement: Figure S8 — Model output in dependence on parameter space. Throughout our analysis we used a reduced system of 12 variants. Given the available data this seemed a good compromise between goodness-of-fit and statistical and computational feasibility. Using a bigger parameter space of 20 variants (A) does result in a slightly improved fit to the transcription data of clone 3D7_AS2, compared to 12 variants (B), whereas a much further reduced system leads to a noticeably less good fit (C). Importantly, in all cases the qualitative switch pathway remains mostly invariant and predicts an initial switch to a number of intermediates and then towards the second dominant variant (which can be seen as significant column biases towards the second variant). Note, as the value of ε is dependent on the dimension of the analysed system we cannot make a direct quantitative comparison between the three models. (0.78 MB TIF) [file ppat.1001306.s008.tif]
