# Supplementary material for: Antigenic Variation in Plasmodium falciparum Malaria Involves a Highly Structured Switching Pattern
Source: PLoS Pathog. 2011 Mar 3;7(3):e1001306. doi: 10.1371/journal.ppat.1001306 (PMC3048365; doi:10.1371/journal.ppat.1001306)
Supplement: Text S1 — Detailed description of the stochastic within-host model. (0.07 MB PDF) [file ppat.1001306.s009.pdf]

Supplementary Information

Within-host model

We developed a simple, discrete-time stochastic model to investigate the effect of switching pathways on the initial dynamics of blood-stage malaria infections, starting off at the first round of the asexual cycle after the liver stage. In the absence of immune control, the growth of a variant is governed by an intrinsic growth rate, assumed to equal for all variants but dependent on host cell availability, and intrinsic switch rates that can differ significantly between variants. The generational growth of parasite variants i at time t, $v_i^{(t)}$, can therefore be given as

$$\bar{v}_i^{(t+1)} = \gamma_i^{(t)} v_i^{(t)} (1 - \omega_i) + \sum_{j \neq i} \gamma_j^{(t)} \omega_j \beta_{ji} v_j^{(t)} \tag{1}$$

with the rate of transcriptional switches per generation, ω_i, and the probabilistic switch bias from variant j to variant i, β_{ji}. Both the switch rate and bias are assumed to be normally distributed around some pre-determined base rates $\tilde{\omega}_i$ and $\tilde{\beta}_{ij}$. The variant's growth rate $\gamma_i^{(t)}$ is also assumed to be normally distributed around a base growth rate γ_0 and under density dependent regulation due to the availability of red blood cells, i.e.

$$\gamma_i^{(t)} = \gamma_0 \left(1 - e^{-\frac{K}{V^{(t)}}}\right), \tag{2}$$

where $V^{(t)}$ is the total parasite load at generation t, i.e. $V^{(t)} = \sum_i v_i^{(t)}$.

The control and removal of parasitised cells is assumed to be multi-factorial, being initially controlled by a non-specific immune response (e.g. fever), NSI, before the variant specific and temporary, cross-reactive immune responses (e.g. antibodies), VSI and CSI, respectively, are initiated after some delay τ. The dynamics of the specific responses are given as:

$$VSI_i^{(t+1)} = VSI_i^{(t)} \left(1 + \pi_i^{VSI(t)}\right)\left(1 - \mu_i^{VSI(t)}\right) \tag{3}$$

$$CSI_i^{(t+1)} = CSI_i^{(t)} \left(1 + \pi_i^{CSI(t)}\right)\left(1 - \mu_i^{CSI(t)}\right) \tag{4}$$

where the first terms on the r.h.s. denote immune expansion in the presence of antigen and the second terms the decay of immune cells in the absence of stimulation. The respecitve rates, π_i and μ_i, are defined as

$$\pi_i^{VSI(t)} = \tilde{\pi}_{VSI} e^{-\frac{\Psi}{v_i^{(t-\tau)}}} \tag{5}$$

$$\pi_i^{CSI(t)} = \tilde{\pi}_{CSI} e^{-\frac{\Psi}{V_i^{(t-\tau)}}} \tag{6}$$

$$\mu_i^{VSI(t)} = \tilde{\mu}_{VSI}\left(1 - e^{-\frac{\Upsilon}{v_i^{(t)}}}\right) \tag{7}$$

$$\mu_i^{CSI(t)} = \tilde{\mu}_{CSI}\left(1 - e^{-\frac{\Upsilon}{V_i^{(t)}}}\right). \tag{8}$$

Here, $\tilde{\pi}$ and $\tilde{\mu}$ are the maximum rates of clonal expansion and decay over a 48h period; Ψ and Υ are the antigen threshold levels necessary for immune stimulation (for simplicity we assume $\Psi = \Upsilon$), and τ is the delay of the adaptive immune response. The specific response VSI_i is triggered only by the presence of antigenic variant i whereas the cross-reactive response is triggered by all variants j that are antigenically similar (related) to variant i, i.e. $V_i = \sum_{j\sim i} v_j$. Without loss of generality we assume that both the variant specific and cross-reactive response have the same growth rate, i.e. $\tilde{\pi}_{VSI} = \tilde{\pi}_{CSI}$, but differ significantly in their decay rate, with $\tilde{\mu}_{VSI} \ll \tilde{\mu}_{CSI}$. With these we can now define the rate of parasite removal by the specific and cross-reactive responses, Γ_i^{ζ}, $\zeta = (VSI, CSI)$, as

$$\Gamma_i^{\zeta} = \alpha_{\zeta}\frac{\zeta_i^{(t+1)}}{\zeta_i^{(t+1)} + \eta_{\zeta} v_i^{(t+1)}}\; e^{-\frac{C+v_i^{(t+1)}}{\zeta_i^{(t+1)}}}. \tag{9}$$

This particular form takes into consideration two important aspects: (i) the removal rate is maximised at very high levels of circulating immune cells, and (ii) the removal rate decreases as the antibody-antigen ratio declines.

The non-adaptive immune response is thought to be immediate (within the 48h-time course of one generation) and is a simple function of parasite load, given as

$$\Gamma^{NSI} = \frac{1}{1 + \left(\frac{C}{1+V^{(t+1)}}\right)^m}, \tag{10}$$

The equation for the dynamics of variant i then becomes

$$v_i^{(t+1)} = \bar{v}_i^{(t+1)}\left(1 - \Gamma_i^{VSI}\right)\left(1 - \Gamma_i^{CSI}\right)\left(1 - \Gamma^{NSI}\right) \tag{11}$$

The important parameters, together with their definition and values are listed in Table 1. Note, the aim of this study was to qualitatively compare different switch mechanism and their influence on the infection dynamics instead of fitting our model to a particular set of data. We therefore did not carry out a comprehensive sensitivity analysis over possible parameter ranges, nor did we aim to make model predictions in terms of the exact parameter constellations that resulted in some best-fit outcome; rather, we devised the model and used parameter values that qualitatively produced biologically realistic behaviours. However, the general results presented here are highly robust to parameter changes.

Table 1: Important model parameters and their biological interpretation.

**parameter**	**definition**	**value**
γ_0	effective base growth rate of parasite	4
$\tilde{\omega}_i$	off-rate of variant i	≤ 0.05
$\tilde{\beta}_{ij}$	switch bias from variant i to j	≤ 1
K	maximum parasite load	10^{13}
$\tilde{\pi}_{VSI}$	max. growth rate VSI	32
$\tilde{\pi}_{CSI}$	max. growth rate CSI	32
$\tilde{\mu}_{VSI}$	decay rate VSI	0.02
$\tilde{\mu}_{CSI}$	decay rate CSI	0.9
α_{VSI}	clearance efficacy VSI	$0 \leq \alpha_{VSI} \leq 1$
α_{CSI}	clearance efficacy CSI	$\alpha_{CSI} \leq \alpha_{VSI}$
